# Generative Models for Medical Image Creation and Translation: A Scoping Review

**DOI:** 10.3390/s26030862

**Published:** 2026-01-28

**Authors:** Haowen Pang, Tiande Zhang, Yanan Wu, Shannan Chen, Wei Qian, Yudong Yao, Chuyang Ye, Patrice Monkam, Shouliang Qi

**Affiliations:** 1 College of Medicine and Biological Information Engineering, Northeastern University, Shenyang 110016, China; haowen.pang@bit.edu.cn (H.P.); 25b903137@stu.hit.edu.cn (T.Z.); shannan_chen@163.com (S.C.); wqian@bmie.neu.edu.cn (W.Q.); 2 School of Integrated Circuits and Electronics, Beijing Institute of Technology, Beijing 100081, China; chuyang.ye@bit.edu.cn; 3 School of Computer Science and Technology, Harbin Institute of Technology, Harbin 150001, China; 4 School of Health Management, China Medical University, Shenyang 110042, China; wuyanan.cmu@vip.163.com; 5 Key Laboratory of Intelligent Computing in Medical Image, Ministry of Education, Northeastern University, Shenyang 110819, China; 6 Department of Electrical and Computer Engineering, Stevens Institute of Technology, Hoboken, NJ 07030, USA; yu-dong.yao@stevens.edu

**Keywords:** generative model, medical image, generative adversarial networks, deep learning

## Abstract

Generative models play a pivotal role in the field of medical imaging. This paper provides an extensive and scholarly review of the application of generative models in medical image creation and translation. In the creation aspect, the goal is to generate new images based on potential conditional variables, while in translation, the aim is to map images from one or more modalities to another, preserving semantic and informational content. The review begins with a thorough exploration of a diverse spectrum of generative models, including Variational Autoencoders (VAEs), Generative Adversarial Networks (GANs), Diffusion Models (DMs), and their respective variants. The paper then delves into an insightful analysis of the merits and demerits inherent to each model type. Subsequently, a comprehensive examination of tasks related to medical image creation and translation is undertaken. For the creation aspect, papers are classified based on downstream tasks such as image classification, segmentation, and others. In the translation facet, papers are classified according to the target modality. A chord diagram depicting medical image translation across modalities, including Magnetic Resonance Imaging (MRI), Computed Tomography (CT), Cone Beam CT (CBCT), X-ray radiography, Positron Emission Tomography (PET), and ultrasound imaging, is presented to illustrate the direction and relative quantity of previous studies. Additionally, the chord diagram of MRI image translation across contrast mechanisms is also provided. The final section offers a forward-looking perspective, outlining prospective avenues and implementation guidelines for future research endeavors.

## 1. Background

In recent years, deep learning has gained widespread prominence in medical image analysis [1,2,3,4,5,6,7] Within the scope of this review, we focus on one of the most compelling applications of deep learning: generative AI in medical imaging, a dynamic and rapidly advancing field of research. The rapid advancement of deep learning and computer vision over the past few decades has had profound implications across a wide range of applications, with the field of medical image generation significantly benefiting from these developments [8,9,10,11,12,13,14].

**Figure 1:**
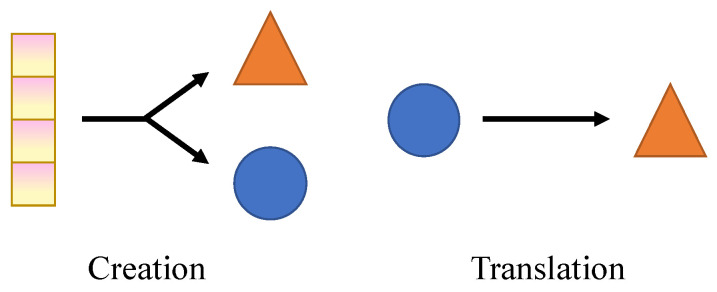
Creation and translation in medical image generation.

In this review, we focus on the application of generative models to medical image creation and modality translation. As shown in Figure 1, image creation aims to generate new images based on potential conditional variables. In deep learning-based image generation, a large dataset of real images is typically used to train the model initially. Subsequently, random noise or conditional inputs are utilized to produce new images. This approach is primarily employed to address challenges in medical imaging, such as data scarcity, insufficient annotations, and severe class imbalances, which are common obstacles in training robust deep learning models [15,16].

Imaging modalities, including Magnetic Resonance Imaging (MRI), Computed Tomography (CT), and Positron Emission Tomography (PET), are commonly used in clinical workflow, each providing unique structural, functional, and metabolic information [17]. Image translation aims to map images from one or more modalities to another while preserving semantic and informational content. The primary goal of medical image translation is to optimize clinical workflows, particularly in situations where traditional imaging methods are impractical due to constraints related to time, labor, or cost [18].

In this review, the terms generation, creation, synthesis, and translation are used with specific distinctions to ensure conceptual clarity. Generation is used as an umbrella term referring to all processes in which generative models are employed to produce medical images. Creation denotes the generation of new images without a direct one-to-one correspondence to existing source images. Synthesis refers to the production of images under explicit clinical or modality-related constraints, emphasizing anatomical plausibility and diagnostic relevance. Translation describes the process of transforming images from one modality or representation into another.

In this context, several key questions guide our investigation: What are the latest advancements in generative models for medical image creation and cross-modality translation? How do different generative model architectures, such as generative adversarial networks (GANs), variational autoencoders (VAEs), and diffusion models, perform in the context of medical imaging, and what are their respective strengths and limitations? Furthermore, how do advanced optimization strategies, including adversarial training, uncertainty modeling, and gradient perturbation, contribute to improving the fidelity, realism, and clinical utility of generated medical images? Finally, what are the primary evaluation metrics used to assess the quality, anatomical accuracy, and clinical applicability of these generated images, and how do these metrics align with the standards of real-world medical practice? By addressing these questions, this review aims to provide a comprehensive understanding of the current state of generative models in medical imaging, highlight emerging trends, and identify areas for future research that could further enhance the capabilities and clinical integration of these powerful technologies.

In this review, we categorize the relevant literature according to its respective applications and thoroughly examine their clinical implications. Furthermore, we explore recent trends and potential future directions in the field. To summarize, the primary contributions of our work are as follows:

1. This review conducts a thorough review of three widely employed generative models: VAEs, GANs, and diffusion models (DMs). We outline algorithms within these generative models that have found extensive applications in the domain of medical image analysis and provide analyses thereof.
2. This review categorizes the applications of generative models in medical image analysis into creation and translation. We present an extensive review of creation methods and classify their downstream applications into three distinct categories: classification, segmentation, and others. We classify translation methods based on the target modality.
3. This review organizes previous studies into categories and offers practical implementation guidelines gleaned from the lessons learned in these works.

**Figure 2:**
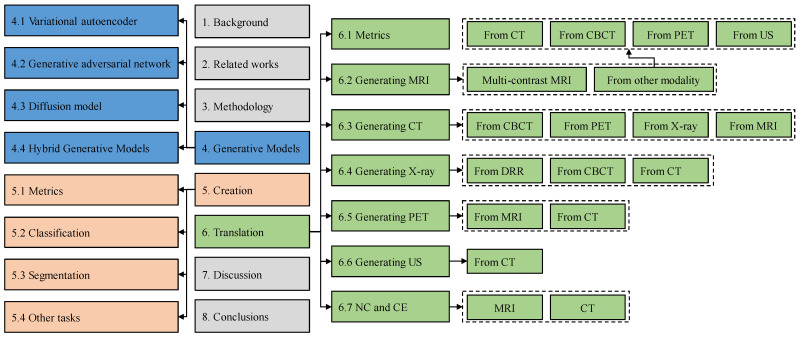
Section organization.

The architecture of this review is shown in Figure 2. In Section 2, we provided a comprehensive comparison with related works. In Section 3, we introduced the search methods for literature and analyzed the trend of the generated model’s publication. In Section 4, we introduced the three most used generation models, VAEs, GANs, DMs, and their variants. In Section 5, we reviewed medical image creation and classified the literature according to different downstream tasks. In Section 6, we reviewed medical image translation and classified the literature according to different target modalities. In Section 7, we summarized the application of generative models in medical image creation and translation and provided implementation guidelines, as well as limitations and future research in this review.

## 2. Related Works

Numerous studies have reviewed the application of generative models in medical image analysis, reflecting the rapid development and growing interest in this field. Yi et al. [19] conducted an early review of the applications of GANs in medical image analysis, covering research up to October 2018. Similar to the work of Yi et al. [19], Kazeminia et al. [20] extended this work by reviewing the applications of GANs in medical image analysis up to October 2019. Their review comprehensively categorized the use of GANs across various tasks, including medical image synthesis, segmentation, reconstruction, detection, denoising, registration, and classification.

Beyond GAN-specific reviews, Wang et al. [18] provided a broader perspective by examining deep learning-based methods for medical image translation, highlighting advancements in cross-modality image synthesis. Dayarathna et al. [17] conducted a comprehensive survey on deep learning-based medical image translation, covering research from 2018 to 2023. Their review focused on the generation of pseudo-CT, MRI, and PET images, providing a detailed overview of synthetic contrasts in medical imaging. Additionally, they summarized the most frequently employed deep learning architectures for medical image synthesis, highlighting key methodologies and their applications in cross-modality image generation.

Additionally, several studies have focused specifically on the role of GANs in medical image augmentation. Chen et al. [21], Goceri et al. [15], and Kebaili et al. [16] conducted a comprehensive and systematic review and analysis of GAN-based medical image augmentation work. Osuala et al. [22] reviewed the application of image synthesis and adversarial networks in the field of cancer imaging. Zhao et al. [23] summarized the application of GAN based on attention mechanisms in tasks such as medical image segmentation, synthesis, and detection.

While these reviews provide valuable insights into the use of GANs and related techniques across diverse medical imaging tasks, there remains a significant gap in the literature. To date, no comprehensive review focuses exclusively on the application of deep learning-based generative models for medical image creation and cross-modality translation. Given the increasing complexity of modern generative architectures, such as diffusion models, VAEs, and transformer-based models, and their transformative potential in medical imaging, a dedicated review in this area is both timely and necessary. This work aims to address this gap by systematically analyzing recent advancements in deep learning-based generative models for medical image creation and translation, with a focus on their clinical relevance, methodological innovations, and future research directions.

## 3. Methodology

This review was conducted and reported in accordance with the Preferred Reporting Items for Systematic Reviews and Meta-Analyses extension for Scoping Reviews (PRISMA-ScR) guidelines. In line with the scope of a scoping review, no formal risk-of-bias assessment was performed. We conducted a rigorous and comprehensive literature search across multiple well-established academic databases, including Web of Science Core Collection, IEEE Xplore Digital Library, ScienceDirect, SpringerLink, and Google Scholar, to ensure the inclusion of high-quality and diverse studies on generative models for medical image creation and translation. Our search strategy was meticulously designed to capture a broad spectrum of relevant research while maintaining precision and relevance. We employed a combination of targeted keywords and phrases, such as “generative models,” “medical image synthesis,” “GAN,” “diffusion models,” and “image translation,” using Boolean operators (e.g., AND, OR) to construct complex search queries that enhanced the sensitivity and specificity of the search. To maintain a contemporary focus, we restricted the search to peer-reviewed articles published between 2018 and 2023, thereby reflecting the latest advancements and emerging trends in the field. Preprint papers were deliberately excluded from our analysis due to the absence of rigorous peer-review processes, ensuring that only validated and credible research findings were considered.

The selection process was conducted in multiple stages to uphold methodological rigor and reduce potential bias. Initially, we performed a broad screening of article titles and abstracts to identify studies potentially meeting our inclusion criteria. This was followed by a comprehensive full-text review of shortlisted articles, which was independently performed by two authors. Studies were included if they explicitly applied generative models to medical image generation, provided detailed descriptions of model architectures and training methodologies, and quantitatively evaluated model performance using established metrics. We excluded articles that lacked methodological transparency, focused solely on theoretical aspects without empirical validation, or addressed non-medical applications. Any disagreements between the two reviewers were resolved through discussion, with unresolved discrepancies adjudicated by a third author to ensure consensus and objectivity. This multi-step screening approach minimized selection bias and enhanced the reliability and reproducibility of our study identification process.

In this review, we exclude the applications of generative models in medical image denoising, reconstruction, super-resolution, registration, etc. This review focuses on modalities primarily used for clinical diagnosis, such as CT, MRI, X-Ray, and PET. These modalities can non-invasively obtain images of entire organs or systems, aiding in clinical diagnosis and treatment monitoring. In this review, we exclude imaging modalities used for studying the microscopic structure of cells and tissues, such as histology and fluorescence microscopy. These modalities are common in pathology, cell biology, and molecular biology research, and are mainly used in laboratory settings to study features at the cellular and molecular levels. They rely on tissue sections and staining techniques, making them suitable for detailed observation at the cellular and tissue levels.

**Figure 3:**
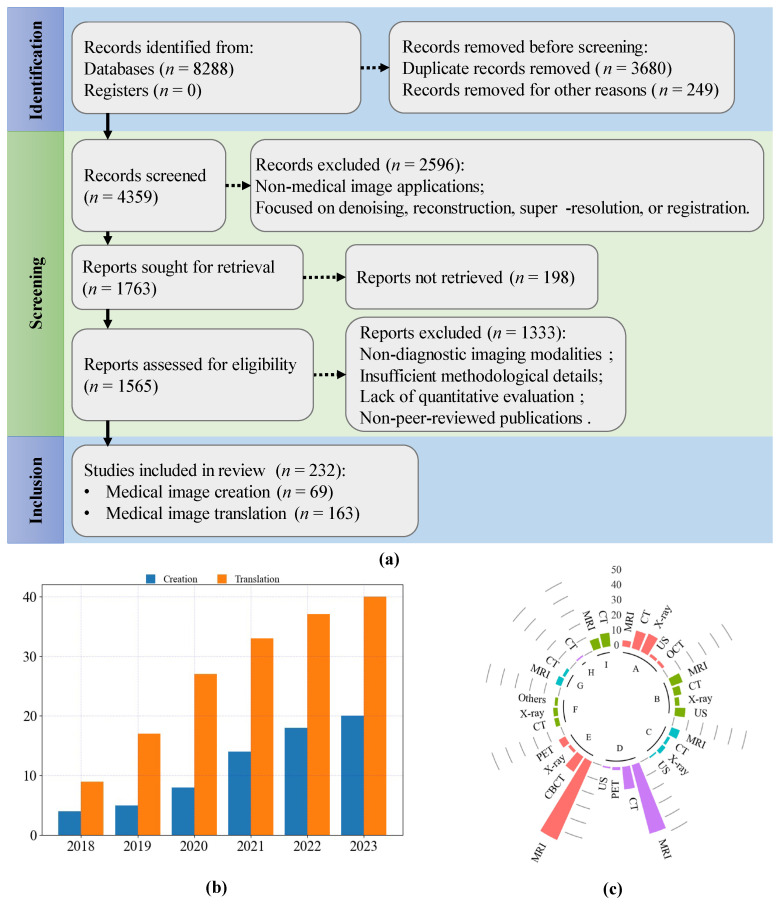
Literature search and analysis. (**a**) The PRISMA flowchart for this review. (**b**) The distribution of articles by year of publication. (**c**) The distribution of articles by task and modality. A: Creation for classification; B: Creation for segmentation; C: Creation for other tasks; D: Translate to MRI; E: Translate to CT; F: Translate to X-ray; G: Translate to PET; H: Translate to Ultrasound; I: Translation with Non- and Contrast Enhanced image.

Through this meticulous and systematic screening, we identified a total of 232 articles that met all predefined inclusion criteria. These articles were incorporated into our review, providing a more extensive and structured analysis compared to prior surveys on the same topic [18,19,21,23,24]. Our systematic approach not only ensures comprehensive coverage of the literature but also facilitates a critical examination of the methodologies, optimization strategies, and clinical implications of generative models in medical imaging. To provide a clear visual representation of our search and selection process, Figure 3 presents a detailed flowchart outlining each stage, from the initial identification of studies to the final inclusion. This figure also illustrates the distribution of the selected articles across different model architectures and medical imaging applications, offering valuable insights into current research trends and gaps in the field.

## 4. Generative Models

Generative models are designed to learn the underlying distribution of a given dataset, in order to generate new data points that resemble the original dataset [25]. These models can generate new data samples that are like the training data, but not identical. Some popular generative models include VAE, GANs, and DMs.

### 4.1. Variational Autoencoder

**Figure 4:**
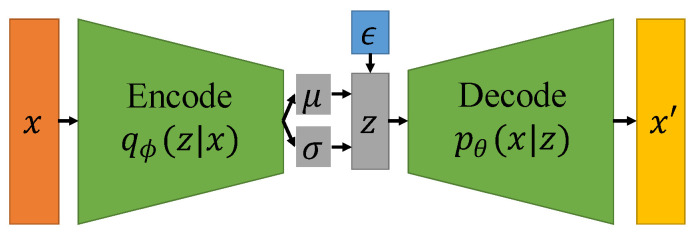
Architecture of VAE.

VAEs [26] have already shown promise in generating complicated nature images [27,28,29] and medical images [30,31]. As shown in Figure 4, the VAE model comprises an encoder network that transforms input data into a latent space representation and a decoder network that reconstructs new data samples from this latent space. Unlike conventional autoencoders, VAEs learn a probabilistic representation of the input data, enabling them to generate novel data samples that closely resemble the original input data [32]. VAEs can generate new medical images that are similar to the original training data, which can be used to augment training datasets and improve the performance of machine learning models. However, the images generated by VAEs tend to be blurrier compared to those generated by other generative models like GANs. This is due to the inherent nature of the VAE’s probabilistic framework, which averages over many possible outputs.

In Figure 4, *x* represents the input image, which is fed into the encoder to obtain two sets of encodings, namely the mean encoding μ and the variance encoding σ. ϵ represents the random noise encoding. By combining the original encoding with the noise encoding after weighted allocation, a new latent code z is obtained, which is then sent to the decoder to reconstruct the original image.

### 4.2. Generative Adversarial Network

**Figure 5:**
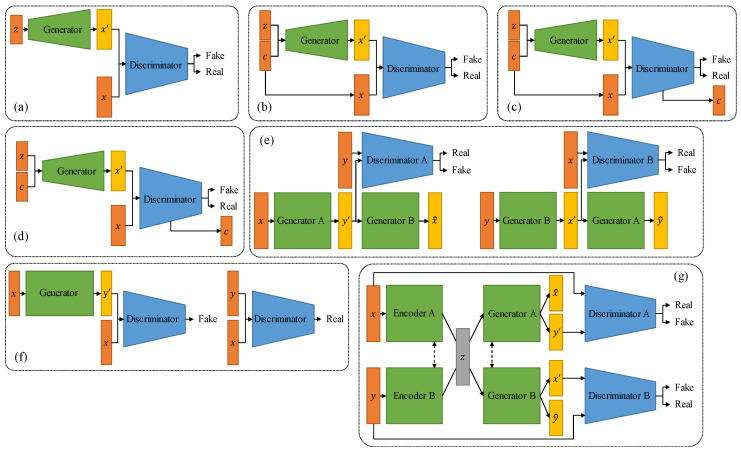
Architecture of GANs. (**a**) Vanilla GAN; (**b**) CGAN; (**c**) ACGAN; (**d**) InfoGAN; (**e**) CycleGAN; (**f**) Pix2Pix; (**g**) UNIT.

As shown in Figure 5a, GAN [33] consists of two neural networks that are trained in an adversarial manner: a generator that generates ‘fake’ data samples that are indistinguishable from the real data, and a discriminator that learns to distinguish between the generated and real data samples [34]. The generator generates new data samples by transforming a low-dimensional input noise vector into a high-dimensional output space that resembles the original data. The discriminator is trained to differentiate between the generated data and the real data. These two networks are optimized through a minimax game framework, wherein the generator aims to create data that can deceive the discriminator, while the discriminator strives to correctly classify the generated data as fake [14].

As shown in Figure 5, variants of GANs have been proposed to address some of the challenges of traditional GANs. For example, conditional GANs (cGANs) add an additional input layer to the generator and discriminator networks, allowing the generator to generate data that satisfies specific conditions, such as class labels or image attributes. Similarly, deep convolutional GANs (DCGANs) use convolutional layers to learn hierarchical features from image data, improving the quality of the generated images. For image translation, Pix2Pix [35] and CycleGAN [36] are the two most commonly used models, and currently, most medical image translation models are modified based on these two models [37,38,39]. GANs and their variants have shown remarkable success in various applications. However, they can be challenging to train and require careful tuning to avoid issues such as mode collapse and instability.

### 4.3. Diffusion Model

**Figure 6:**
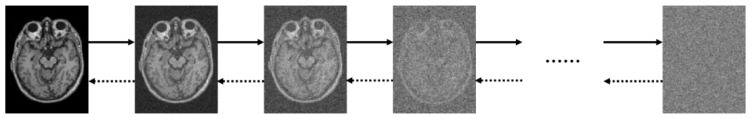
Architecture of the diffusion model.

Diffusion models have been applied to various fields of image generation [34]. As shown in Figure 6, the diffusion model is a type of probability generation model that gradually adds noise to the data to break the structure of the data, and then learns a corresponding reverse process to denoise, thereby learning the distribution of the original data. The forward diffusion process incrementally adds noise to the input data, progressively increasing the noise level until the data is entirely transformed into pure Gaussian noise. This process systematically disrupts the underlying structure of the data distribution. The reverse diffusion process, often referred to as denoising, is then employed to reconstruct the original data structure from the perturbed distribution. This step effectively reverses the degradation introduced by the forward diffusion process. As a result, a highly flexible and tractable generative model is achieved, capable of accurately modeling complex data distributions starting from random noise [34].

Recently, diffusion models and their variants have been applied to medical image analysis, including medical image creation [40,41], translation [42], reconstruction [43,44], denoising [45], registration [46], classification [47], and segmentation [48,49].

### 4.4. Hybrid Generative Models

In addition to standalone generative paradigms, recent studies have increasingly explored hybrid generative models that integrate complementary mechanisms from multiple frameworks. These hybrid approaches aim to mitigate the inherent limitations of individual models while leveraging their respective strengths. Typical hybrid designs include diffusion–GAN or diffusion–autoencoder hybrids that employ diffusion processes for global structure modeling and adversarial losses for enhancing local realism [50]. Compared with standalone generative models, hybrid approaches often demonstrate improved perceptual quality and training stability, particularly in scenarios involving limited annotated data [51]. However, these advantages come at the cost of increased architectural complexity, higher computational requirements, and more challenging optimization procedures [52,53].

### 4.5. Training Stability and Computational Requirements

In summary, VAEs generally exhibit stable and well-behaved training dynamics with relatively modest computational requirements, but often suffer from limited image fidelity. In contrast, GANs are capable of producing high-quality images but are known to be sensitive to hyperparameter settings and prone to training instability, which may require careful optimization and increased computational overhead. Diffusion-based models demonstrate superior robustness during training and strong generation performance, albeit at the cost of substantially higher computational complexity and longer training and inference times. Hybrid approaches aim to balance these trade-offs by combining complementary strengths of different paradigms, though they often introduce additional architectural complexity.

## 5. Creation

Due to the inherent structural complexity and large parameter scale of deep learning models, a significant amount of labeled data is typically required for their effective training. The acquisition of labeled medical image data heavily depends on the subjective expertise and professional judgment of radiologists [54]. Additionally, it is susceptible to issues related to image quality, leading to significant challenges such as data scarcity, insufficient annotations, and pronounced class imbalances. These limitations significantly hinder the broader adoption of deep learning models and represent a critical obstacle in the development of deep learning-based medical diagnostic systems [15].

Medical image data augmentation serves as a technique employed to augment the quantity and diversity of available medical images for training machine learning models [16]. Traditional data augmentation techniques include methods such as image quality enhancement, adjustments to brightness or contrast, and geometric transformations like rotation, scaling, and deformation [15]. The ascendancy of deep learning-based generative models in the generation of data has garnered substantial attention. Within the domain of medical image analysis, the utilization of deep learning-based generative models for the generation of medical image data assumes paramount significance. This approach can simulate a substantial volume of challenging-to-obtain medical image data, effectively mitigating the adverse impact of data scarcity on the domain of medical image analysis [22].

**Figure 7:**
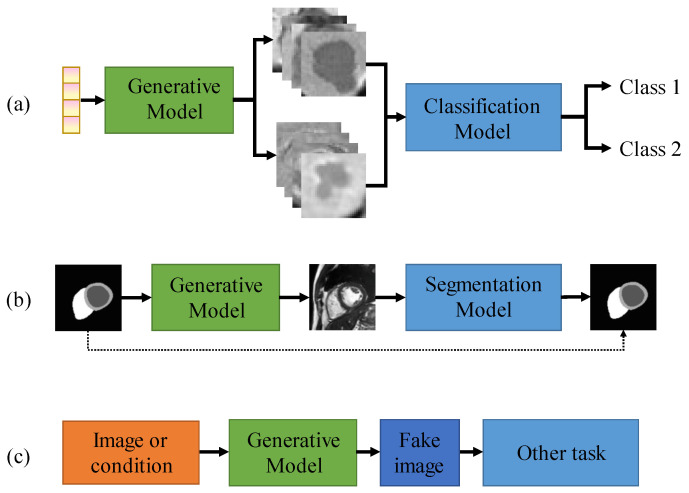
Creation for downstream tasks: (**a**) creation for classification; (**b**) creation for segmentation; (**c**) creation for other tasks.

In this section, we summarize the application of generative models in medical image creation. We review the literature based on downstream tasks, namely classification tasks, segmentation tasks, and other tasks. As shown in Figure 7a, it is used for creating medical image data for classification tasks. Specifically, various classes of medical images are created from random noise, and then the created data is used to train a classification model. Figure 7b is used for creating medical image data for segmentation tasks. Medical images are created from segmentation masks, and then the created data and masks are used to train a segmentation model. Figure 7c is used for creating medical image data for other downstream tasks, such as regression, object detection, and survival prediction.

### 5.1. Metrics of Medical Image Creation

In order to verify the performance of the proposed medical image creation method, it is necessary to use metrics to evaluate the similarity between the generated image. Table 1 lists several commonly used image similarity evaluation metrics. $P_{r}$ denotes the real image distribution, $P_{g}$ denotes the generated image distribution. $p_{M}\left(y|x\right)$ denotes the label distribution of x as predicted by M, and $p_{M}\left(y\right)=\int p_{M}\left(y|x\right)dP_{g}$. $p_{M}\left(y^{*}\right)=\int p_{M}\left(y|x\right)dP_{r}$ is the marginal label distribution for the samples from the real data distribution. $\Gamma (P_{r},P_{g})$ denotes the set of all joint distributions (i.e., probabilistic couplings) whose marginals are respectively $P_{r}$ and $P_{g}$, and $d(x_{r},x_{g})$ denotes the base distance between the two samples.

The Inception Score (IS) uses a pre-trained Inception-v3 model to compute the KL-divergence between the predicted class distributions of generated images and their overall diversity [55]. A higher IS indicates better image quality and diversity. The Mode Score (MS) adds a measure of similarity between the probability distributions of generated samples and real samples based on the IS. Kernel Maximum Mean Discrepancy (MMD) quantifies the difference between the probability distributions of generated samples and real samples using a fixed kernel function. Wasserstein Distance (WD) serves as a metric to evaluate the similarity between two distributions, where a smaller WD indicates greater similarity. Fréchet Inception Distance (FID) computes the Wasserstein-2 distance between the distributions of feature vectors derived from generated and real images, utilizing a pre-trained Inception-v3 model for feature extraction [55]. A lower FID indicates better image quality.

### 5.2. Classification

In recent years, there have been notable developments in medical image classification techniques, driven by advancements in deep learning algorithms [56]. However, several challenges and limitations remain. First, acquiring large-scale medical image datasets is often difficult due to privacy concerns, limited accessibility, and ethical constraints. Second, training medical image classification models necessitates the involvement of expert radiologists, pathologists, or clinicians to manually annotate the images with appropriate labels or categories. This annotation process is not only labor-intensive but also requires specialized expertise, creating significant barriers to effectively training deep learning models. Third, medical datasets frequently exhibit class imbalance, where certain disease categories are underrepresented compared to others, further complicating model training and evaluation [57]. Detecting rare diseases or conditions with limited training samples poses a challenge, as models tend to favor the majority classes during training.

In this section, we undertake a comprehensive review of the pertinent literature about medical image creation for classification. We compile essential information from the literature and present it in Table 2.

Table 2 provides a comprehensive overview of 27 literature sources, with most of them being based on GAN. Among these 27 sources, the highest number of publications is focused on the chest, with 14 of them specifically targeting chest-related studies. The most common application is in the generation of X-ray and CT images. Additionally, most of the literature is based on 2D models, possibly due to limitations in GPU memory.

Pesteie et al. [30] introduced a variational generative model to learn the probability distribution of image data conditioned on latent variables and corresponding labels. The trained model is employed to generate new images for data augmentation. The efficacy of this approach is demonstrated through its application to ultrasound images (US) of the spine and brain MRI. This model resulted in a notable enhancement in the accuracy of the classification task.

Salehinejad et al. [61] proposed a DCGAN to create chest X-rays. They utilized both real and created images to train a model for the detection of pathology across five classes of chest X-rays. A comparative analysis of DCNNs trained with a mixture of real and created images revealed that the model outperformed its counterparts trained exclusively with real images.

Pan et al. [40] proposed an image creation framework based on a diffusion model utilizing a Swin-transformer-based network. This model encompasses a forward Gaussian noise process and a reverse process employing the transformer-based diffusion model for denoising. COVID-19 classification models were trained using real images, created images, and combinations of both.

Applying generative model–based data augmentation to medical image classification has been extensively explored as a strategy to mitigate data scarcity and class imbalance. Existing studies suggest that generative models can approximate the underlying data distribution and produce samples that resemble real medical images, which may enhance classification performance when incorporated into the training set [83].

In particular, generative augmentation has been shown to be beneficial in scenarios involving limited training data or severe class imbalance [84]. Several studies report measurable improvements in accuracy and AUC when generated samples are used to augment minority classes, especially for rare disease categories or small-scale datasets. By increasing the effective sample size and improving class balance, generative models can help reduce bias toward majority classes during training. Beyond dataset expansion, generative models can introduce controlled intra-class variability by creating samples with diverse appearances, shapes, or textures. This diversity may facilitate the learning of more robust and discriminative features, thereby improving generalization to unseen data. Such benefits are more likely to be observed when the generated images are anatomically consistent and preserve clinically relevant label information. However, the effectiveness of generative model–based data augmentation is highly task- and data-dependent. Empirical evidence indicates that performance gains are not guaranteed. Several studies report marginal improvements or even performance degradation when generated images contain subtle artifacts, blur class-discriminative structures, or introduce distributional shifts relative to real data. These issues are particularly pronounced in fine-grained classification tasks, where minor anatomical differences carry critical diagnostic significance. Moreover, generative augmentation tends to offer limited benefits when sufficient real training data are available. In such cases, classifiers may overfit to generated patterns rather than learning robust representations from authentic clinical images. Importantly, increasing the proportion of generated data does not necessarily result in monotonic performance improvements; multiple studies have observed performance saturation or decline when generated samples dominate the training set.

Overall, generative model–based data augmentation should be viewed as a complementary tool rather than a universal solution for medical image classification. Its effectiveness depends on the quality of the generated samples, the characteristics of the target task, and the balance between generated and real data. Careful empirical validation is therefore essential to determine when generative augmentation provides meaningful performance gains and when it may compromise classification reliability.

### 5.3. Segmentation

Developing a medical image segmentation model necessitates the expertise of radiologists or clinical professionals to manually annotate the images, thereby establishing ground truth data that serves as a reference for training and evaluating the segmentation model [85]. Manual annotation is a time-intensive, subjective process reliant on expert knowledge, rendering the task of constructing extensive and diverse datasets a formidable endeavor.

The generative models provide the images and masks required for training medical image segmentation models by converting masks into generated images. This approach significantly mitigates the demand for annotated data. In this section, we embark on an exhaustive review of the pertinent literature, which we present comprehensively in Table 3.

Table 3 offers a comprehensive overview of 26 literature sources, with the majority of them centering on GAN. Similar to the emphasis on data creation for classification tasks, a significant number of publications focus on chest and lung-related topics. The most prevalent applications involve the generation of X-ray, CT, and ultrasound images. As with creation for classification, most of the literature is based on 2D models.

Guo et al. [110] introduced a confidence-guided generation of anatomic and molecular MR image networks (CG-SAMR) that enables the generation of data by leveraging lesion contour information into multi-modal MR images. Additionally, they extended the proposed architecture to support training with unpaired data. The generated data proves valuable for data augmentation, especially in the context of images containing pathological information related to gliomas.

Zhang et al. [94] presented an improved Dense GAN for data augmentation. They harnessed the power of Dense GAN to generate CT images, facilitating effective semi-supervised segmentation.

Amirrajab et al. [95] proposed a method for generating cardiac MR images with plausible heart shapes and appearances to create labeled data. The approach dissects image generation into two tasks: label deformation and label-to-image translation. Label deformation is achieved through latent space interpolation within the VAE model, while label-to-image translation is accomplished using a conditional GAN.

### 5.4. Other Tasks

In addition to classification and segmentation tasks, there are other tasks in the field of medical image analysis, such as regression, object detection, and survival prediction. There are currently many proposed data augmentation methods based on generative models for these tasks. We compile essential information from the literature and present it in Table 4. In addition, we also collected some literature without specified downstream tasks, and they are all listed in Table 4.

Han et al. [112] introduced a 3D Multi-Conditional GAN (MCGAN) to generate nodules on lung CT images to enhance sensitivity in object detection. The MCGAN incorporates two discriminators: the context discriminator and the nodule discriminator. The results demonstrate that 3D CNN-based detection achieves increased sensitivity for nodules of any size or attenuation at fixed false positive rates, effectively addressing the scarcity of medical data by leveraging MCGAN-generated realistic nodules.

Kamli [113] proposed a Synthetic Medical Image Generator (SMIG) with the primary aim of generating MRI using a GAN to provide anonymized data. Furthermore, to predict the growth of glioblastoma multiform tumors, the authors developed a tumor growth predictor. The authors emphasized the significance of employing data generated by SMIG. Despite the limited dataset size available from the public dataset, the results demonstrate valuable accuracy in predicting tumor growth.

Li et al. [121] introduced DeepAnat, a method to generate high-quality T1 images from diffusion MRI and to perform brain segmentation on generated T1 images and assist co-registration using generated T1 images. This study underscores the advantages and practical feasibility of creating medical images to support various diffusion MRI data analyses and their utility in neuroscientific applications.

## 6. Translation

Medical image modality translation refers to the process of converting images from one or more modalities into different modalities [122], such as transforming from CT to MRI or from T1 and T2 to FLAIR. Medical image modality translation proves invaluable when medical imaging data is scarce or when patients cannot undergo specific imaging modalities due to medical or technical constraints. Modality translation empowers medical professionals and researchers to access more comprehensive information about a patient’s medical condition, enhancing the accuracy of diagnosis and treatment planning [18].

Conventional methods entail the utilization of models with predefined rules to effectuate the conversion of images from one modality to another. These models necessitate manual parameter adjustments to achieve optimal performance and are often tailored to specific applications, contingent upon the distinctive characteristics of the involved imaging modality [18]. Consequently, numerous intricate and application-specific techniques have been developed. However, these methods confront challenges when the two imaging modalities provide disparate information, rendering the establishment of an effective model a formidable undertaking.

In tandem with the advancement of deep learning, an increasing array of modality translation methods grounded in deep learning principles has emerged. Deep learning-based generative models, exemplified by GANs and diffusion models, have exhibited tremendous potential in the domain of medical image modality translation [20]. They excel by acquiring the capability to learn the mapping between different modalities and generating high-quality images.

**Figure 8:**
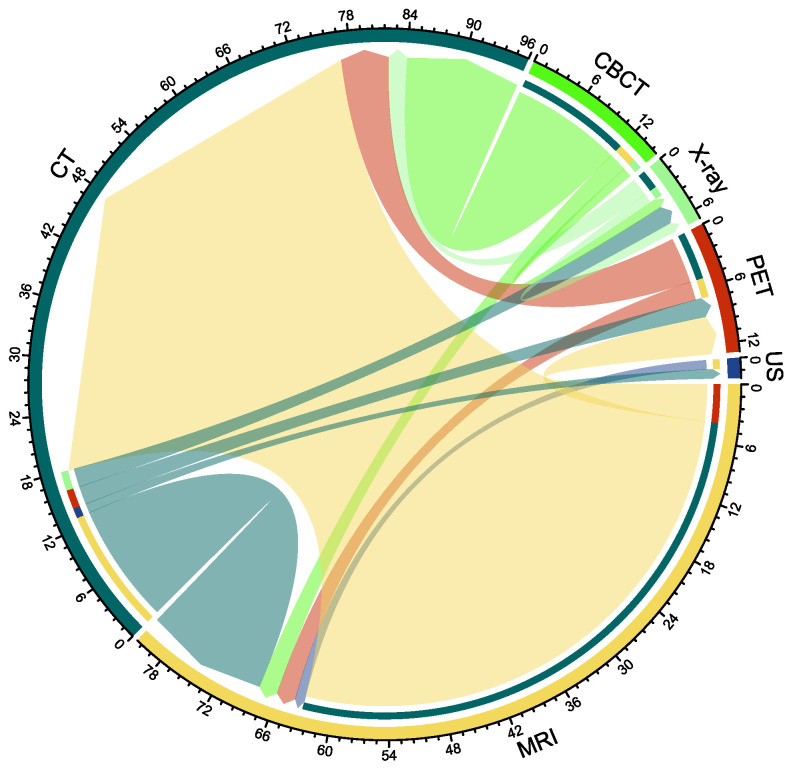
Chord diagram of medical image translation cross-modalities of MRI, CT, CBCT, X-ray, PET, and US.

In this section, we classify the collected modality translation literature according to the target modality. In Figure 8, the literature quantity is shown for translations between CT, MRI, CBCT, X-ray, PET, and ultrasound images. The number of studies of the six source modalities is in the order of MRI (63), CT (18), CBCT (15), PET (7), X-ray (3), and US (1). The number of studies of the six targeted modalities is in the order of CT (78), MRI (18), PET (6), X-ray (4), and US (1). There are four kinds of translations worthy of attention. The first is the translation from MRI to CT (59 studies), primarily focusing on dose calculation for MRI-guided radiation therapy. The second is the translation from CT to MRI (13 studies), primarily aiming for more accurate segmentation. The third is the translation from CBCT to CT, with the main objective of 12, primarily serving the objectives of image denoising and dose calculation. The fourth is to translate PET to CT specifically for attenuation correction.

In addition, the translation between non-contrast images and contrast images has also been a research hotspot in recent years, and we will separately organize them in Section 6.7.

### 6.1. Metrics of Medical Image Translation

In order to verify the performance of the proposed modality translation method, it is necessary to use metrics to evaluate the similarity between the synthesized image and the real image. Table 5 lists several commonly used image similarity evaluation metrics.

Mean Absolute Error (MAE) provides a straightforward, easy-to-interpret measurement of error. It gives equal weight to all errors, regardless of their magnitude, making it less sensitive to outliers. Mean Squared Error (MSE) gives a more significant penalty to large errors compared to MAE, which can be desirable in some contexts. It is also widely used and mathematically convenient. Peak Signal-to-Noise Ratio (PSNR) is based on MSE and shares some of its limitations [123]. It does not always align with human perception, especially for complex images or artifacts like blockiness. Structural Similarity Index (SSIM) is designed to measure the similarity between two images in terms of luminance, contrast, and structure, which aligns better with human perception [124]. It is often considered more accurate than PSNR for evaluating image quality. In summary, MAE and MSE are simple and widely used metrics that are easy to compute but may not always align with human perception. PSNR is also widely used and easy to interpret, but may not correlate well with perceptual quality. SSIM, on the other hand, is more aligned with human perception but can be more computationally expensive. Choosing the right metric depends on the specific requirements of the application and the aspects of image quality that are most important.

In the equations in Table 5, $x_{i}$ and $y_{i}$ are the pixel values of position i in the image x and y, respectively. MAX is the maximum possible pixel value. $\mu_{x}$ and $\mu_{y}$ are the mean value of image x and y, respectively. $\sigma_{x}^{2}$ and $\sigma_{y}^{2}$ are the variance of image x and y, respectively.$\;\sigma_{xy}$ is the covariance of image x and y. $C_{1}$ and $C_{2}$ are constants.

### 6.2. Generating MRI

#### 6.2.1. Multi-Contrast MRI Translation

MRI stands as a non-invasive medical imaging technique utilizing a potent magnetic field and radio waves to generate intricate images of internal organs and tissues within the human body [38]. Various MRI modalities, including T1-weighted (T1w), T2-weighted (T2w), Diffusion-Weighted Imaging (DWI), Magnetic Resonance Angiography (MRA), and Fluid-Attenuated Inversion Recovery (FLAIR), offer distinctive characteristics and applications. In tumor analysis, T1-weighted scans excel at differentiating gray and white matter in brain images, while T2-weighted images enhance the contrast between fluid and cortical tissue. FLAIR (Fluid-Attenuated Inversion Recovery) sequences are particularly effective in suppressing cerebrospinal fluid signals, improving lesion visibility. T1 contrast-enhanced (T1ce) images are valuable for delineating tumor regions in brain scans. Magnetic Resonance Angiography (MRA) is primarily used to evaluate vascular anatomy and detect abnormalities that may predispose to hemorrhages. Proton density (PD) images are widely utilized in radiology for inferring water content, aiding in lesion classification, and multispectral segmentation. The integration of these multimodal MRI scans provides complementary information, with each modality offering unique insights into the body’s internal structures and functions. Together, they deliver a comprehensive assessment of the patient’s condition [38].

In some cases, it may be difficult to collect complete modalities for medical image analysis due to factors such as the cost of long-term examinations and uncooperative patients, particularly children and the elderly [125]. In such situations, synthesizing missing or damaged modalities using successfully acquired modalities can improve the availability of diagnosis-related images and enhance analysis tasks such as classification and segmentation. In recent years, with the development of deep learning based generative models, there has been an increasing amount of work on the translation between MRI modalities. Table 6 lists essential information about these works.

**Figure 9:**
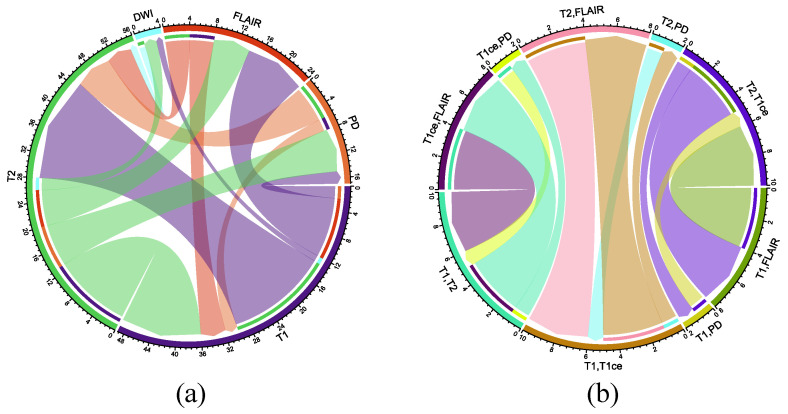
Chord diagram of MRI image translation across contrast mechanisms. (**a**) Single-to-single translation; (**b**) multi-to-multi translation.

In Figure 9a, based on the number of studies, the primary translations can be ranked as T1-to-T2 (17), T2-to-T1 (13), T1-to-FLAIR (10), T2-to-PD (7), T2-to-FLAIR (6), PD-to-T2 (6), FLAIR-to-T1 (4), FLAIR-to-T2 (4), T1-to-PD (2), PD-to-T1 (2), T2-to-DWI (2), and DWI-to-T2 (1). In Figure 9b, according to the number of studies, the main translations can be ranked as (T1, FLAIR)-to-(T2, T1ce) (4), (T1, T1ce)-to-(T2, FLAIR) (4), (T1, T2)-to-(T1ce, FLAIR) (4), (T1ce, FLAIR)-to-(T1, T2) (4), (T2, FLAIR)-to-(T1, T1ce) (4), and (T2, T1ce)-to-(T1, FLAIR) (4). The fundamental objective of MRI image translation across contrast mechanisms is to avoid the acquisition of actual scans and provide the unavailable MRI modality necessary for diagnosis and treatment.

In most cases, as the number of source modalities increases, the model’s performance tends to improve. On the same dataset, the performance of multi-to-single translation is superior to single-to-single translation. This is because more modalities can provide complementary information to each other, leading to a more realistic target modality. However, when the number of source modalities remains the same, and the number of target modalities increases, no fixed trend in performance has been observed.

As shown in Table 6, there are several widely used datasets in cross-modality MRI translation, such as IXI, BraTS, and ISLES. The IXI dataset comprises nearly 600 MRIs obtained from normal and healthy subjects. The MRI acquisition protocol for each subject includes a comprehensive set of sequences: T1, T2, PD, MRA, and DWI. These data have been collected across three different hospitals. The Brain Tumor Segmentation (BraTS) dataset is a widely recognized and frequently used collection of medical images specifically designed for brain tumor research, particularly in the field of medical image analysis and machine learning. The dataset includes multimodal brain MRI scans, typically comprising T1, T1ce, T2, and FLAIR images. The Ischemic Stroke Lesion Segmentation (ISLES) challenge is dedicated to evaluating infarct segmentation in both acute and sub-acute stroke cases, leveraging multimodal MRI data. The inaugural ISLES challenge, held in 2015, was divided into two sub-challenges: Sub-acute Stroke Lesion Segmentation (SISS) and Stroke Perfusion Estimation (SPES). SISS aimed to segment subacute stroke lesions using conventional post-stroke MRI sequences, including T1, T2, FLAIR, and DWI. The ISLES 2018 challenge focused on predicting infarct core delineation in DWI using CT perfusion data. The primary objective of the ISLES 2022 challenge is to segment stroke lesions from DWI, ADC, and FLAIR sequences, with a dataset comprising 400 cases.

Currently, most methods are based on GANs, and most of these methods utilize 2D network architecture, possibly due to memory constraints. Furthermore, algorithms that require paired data for training are more prevalent than those that can use unpaired data because paired images can provide better supervision, leading to improved model performance.

Salman et al. [38] proposed pGAN and cGAN for multi-contrast MRI translation, leveraging conditional GANs. The proposed approach preserves intermediate-to-high frequency details through an adversarial loss, providing enhanced synthesis performance using pixel-wise and perceptual losses for registered multi-contrast images and a cycle-consistency loss for unregistered images.

Zhou et al. [133] introduced a Hybrid-fusion Network (Hi-Net) for multi-modal MRI translation, which learns a mapping from multi-modal source images to target images. In Hi-Net, a modality-specific network is employed to learn representations for each individual modality, and a fusion network is utilized to learn the common latent representation of multi-modal data. Subsequently, a multi-modal translation network is designed to densely combine the latent representation with hierarchical features from each modality, acting as a generator to synthesize the target images.

Muzaffer et al. [42] proposed SynDiff, employing an adversarial diffusion model for multi-contrast MRI translation. To capture a direct correlate of the image distribution, SynDiff utilizes a conditional diffusion process that progressively maps noise and source images onto the target image. For efficient and accurate image sampling during inference, large diffusion steps are taken with adversarial projections in the reverse diffusion direction.

#### 6.2.2. Generating MRI from Other Modalities

In this section, we summarize the papers on the translation from non-MRI modalities to MRI. The number of papers on CT-to-MRI is the highest. Table 7 lists essential information about these works.

Wang et al. [173] introduced a bidirectional learning model, denoted as dual contrast CycleGAN (DC-CycleGAN), designed to synthesize MRI from CT. Specifically, a dual contrast loss is incorporated into the discriminators to indirectly establish constraints between real source and synthetic images. This is achieved by leveraging samples from the source domain as negative samples, enforcing the synthetic images to diverge significantly from the source domain. Additionally, cross-entropy and the structural similarity index (SSIM) are integrated into the DC-CycleGAN to consider both the luminance and structure of samples during image translation.

Lei et al. [175] proposed a method for generating MRIs with superior soft-tissue contrast from CBCT images to aid CBCT segmentation. The entire segmentation process comprises three major steps. Firstly, CycleGAN is utilized to estimate a synthetic MRI (sMRI) from CBCT images. Secondly, a deep attention network is trained based on sMRI and its corresponding manual contours. Finally, segmented contours for a query patient are obtained by feeding the patient’s CBCT images into the trained sMRI estimation and segmentation model.

Bazangani et al. [178] proposed a separable convolution-based Elicit Generative Adversarial Network (E-GAN). The architecture can generate a 3D T1-weighted MRI corresponding to FDG-PET.

### 6.3. Generating CT

CT is a potent medical imaging technique that employs X-ray technology and computer processing to generate cross-sectional images of the human body. CT delivers highly detailed cross-sectional views of internal structures, allowing for precise examination and analysis of anatomical features, organs, and bones [180]. CT scanning plays a pivotal role in diagnosing a wide array of medical conditions, including traumatic injuries like fractures and internal hemorrhaging, as well as the detection and assessment of tumors, vascular disorders like aneurysms and blockages, lung diseases such as pneumonia and cancer, and neurological disorders like strokes, brain tumors, and related conditions [181,182].

However, it is imperative to consider potential risks associated with CT scans due to their use of ionizing radiation, particularly when repeated imaging is necessary [183]. Furthermore, CT serves as the primary imaging modality for radiation therapy, as it provides essential electron density data for dose calculations. While MRI excels in visualizing soft tissues and tumors, it lacks the tissue attenuation information required for accurate dose calculations in radiation therapy. The utilization of generative models to translate MRI into CT images is pivotal in enabling MRI-only radiotherapy, which can yield cost savings, reduce patient radiation exposure, and eliminate registration errors associated with using two distinct imaging modalities [184].

Cone Beam Computed Tomography (CBCT) represents an advanced medical imaging technique widely applied in fields such as dentistry and maxillofacial radiology [185]. CBCT employs a cone-shaped X-ray beam and a specialized detector to produce high-resolution, three-dimensional images of specific regions of interest within the human body, primarily focusing on the craniofacial area. Notably, CBCT offers the advantage of lower radiation doses, enhancing patient safety, while still delivering exceptional image clarity for detailed visualization of anatomical structures like teeth, bones, and soft tissues. However, CBCT does have inherent limitations, including lower contrast for soft tissues and reduced spatial resolution compared to conventional CT. Additionally, CBCT is more susceptible to metal artifacts, potentially compromising image quality when scanning patients with dental restorations or implants. Therefore, the development of generative models to translate CBCT images into CT is of considerable significance [186].

In this section, we provide a comprehensive summary of research papers related to the translation from various imaging modalities, including CBCT, MRI, PET, and X-Ray, into CT. Table 8 lists essential information about these works.

Zhang et al. [186] decomposed CBCT-to-CT translation into artifact reduction and intensity correction. They proposed a Multimodal Unsupervised Representation Disentanglement (MURD) learning framework that disentangles content, style, and artifact representations from CBCT and CT images in the latent space. MURD can synthesize different forms of images by recombining disentangled representations. Additionally, they introduced a multipath consistency loss to enhance structural consistency in synthesis and a multidomain generator to improve synthesis performance.

Dong et al. [196] proposed a 3D CycleGAN framework to synthesize CT images from non-attenuation corrected PET (NAC PET). The method learns a transformation that minimizes the difference between sCT, generated from NAC PET, and true CT. It also learns an inverse transformation such that the cycle NAC PET image generated from the sCT is close to the true NAC PET image.

Zhou et al. [248] proposed a multimodality MRI synchronous construction-based deep learning framework from a single T1-weighted image for MRI-guided radiation therapy (MRIgRT) synthetic CT (sCT) image generation. The network is primarily based on a GAN with sequential subtasks of intermediate synthetic MRI generation and joint sCT image generation from the single T1 MRI. It comprises a multitask generator and a multibranch discriminator, where the generator consists of a shared encoder and a split multibranch decoder.

### 6.4. Generating X-Ray Image

In this section, we provide a comprehensive summary of research papers related to the translation from various imaging modalities into X-rays. A summarized overview of these works is presented in Table 9, highlighting essential information for reference.

Yuen et al. [252] introduced a CT-based Chest X-ray (CXR) synthesis framework, named ct2cxr, for data augmentation in pneumonia classification. Leveraging GANs and a customized loss function tailored for model training, the approach aims to preserve target pathology and maintain high image fidelity. The results indicate that CXR images generated through style mixing enhance the performance of general pneumonia classification models. Evaluation on a COVID-19 dataset demonstrates similar improvements over baseline models.

Huang et al. [250] proposed a sigmoid-based intensity transform, utilizing the nonlinear optical properties of X-ray films, to enhance image contrast in synthetic cephalograms generated from 3D volumes. Super-resolution deep learning techniques are explored to improve image resolution. For low-dose purposes, Pix2pix is introduced for 2D cephalogram synthesis directly from two cone-beam projections. An efficient automatic landmark detection method for synthetic cephalograms is proposed, combining LeNet5 and ResNet50.

Shen et al. [253] proposed a strategy for obtaining X-ray projection images at novel view angles without the need for actual projection measurements. Specifically, a Deep Learning-based Geometry-Integrated Projection Synthesis (DL-GIPS) framework is proposed for generating novel-view X-ray projections. The deep learning model extracts geometry and texture features from a source-view projection, then performs geometry transformation on the extracted features to accommodate the change in view angle. In the final stage, the X-ray projection in the target view is synthesized from the transformed geometry and shared texture features via an image generator.

### 6.5. Generating PET Image

Positron Emission Tomography (PET) is a powerful medical imaging technique. It is based on the principle of detecting and visualizing the distribution of positron-emitting radionuclides within the body [254]. PET imaging has a wide range of clinical applications. PET is used to detect and stage various types of cancers by highlighting areas with increased metabolic activity. PET is valuable in studying brain function and diagnosing conditions such as Alzheimer’s disease, Parkinson’s disease, and epilepsy. PET can assess blood flow and myocardial viability, helping in the evaluation of heart conditions, including coronary artery disease and myocardial infarction. PET is used to identify sites of infection or inflammation in the body, which can aid in the diagnosis and monitoring of infectious diseases and inflammatory disorders. PET allows scientists to study various physiological processes, develop new drugs, and better understand diseases at the molecular level. PET provides functional and metabolic information, complementing the structural information obtained from techniques like CT and MRI [255]. It can detect diseases at an early stage when structural changes may not yet be apparent. PET has high sensitivity and specificity, making it a valuable tool for accurate disease detection and treatment monitoring [256].

Of course, PET also has some limitations. PET involves exposure to ionizing radiation due to the use of radiopharmaceuticals. It requires specialized equipment and trained personnel. PET scans may be expensive compared to some other imaging modalities. So, there is currently some work dedicated to converting other commonly used medical image modalities, such as MRI and CT, into PET. In this section, we provide a comprehensive summary of research papers related to the translation from CT or MRI into a PET image. A summarized overview of these works is presented in Table 10, highlighting essential information for reference.

Hu et al. [254] introduced a 3D end-to-end translation network named Bidirectional Mapping GAN (BMGAN) for brain MR-to-PET translation, effectively utilizing image contexts and latent vectors. The proposed bidirectional mapping mechanism is designed to embed the semantic information of PET images into the high-dimensional latent space. Furthermore, the architecture includes a 3D Dense-UNet generator and hybrid loss functions to enhance the visual quality of cross-modality synthetic images.

Ben-Cohen et al. [256] combined a fully convolutional network (FCN) with a conditional GAN to simulate PET data from input CT data. From a clinical perspective, such solutions may facilitate lesion detection and drug treatment evaluation in a CT-only environment, potentially reducing the need for more expensive and radioactive PET/CT scans.

### 6.6. Generating Ultrasound Image

Ultrasound imaging is a non-invasive medical imaging technique that uses high-frequency sound waves to create real-time, dynamic images of the internal structures of the human body [260]. These images, known as ultrasound scans or ultrasound images, are valuable in medical diagnosis, monitoring pregnancies, and guiding various medical procedures.

Ultrasound imaging does not involve radiation or invasive procedures. It provides dynamic, real-time images, making it suitable for observing movement and function. Ultrasound is safe for pregnant women, infants, and individuals with contraindications to other imaging methods. Ultrasound machines come in various sizes, including handheld devices, making them highly portable for use in different clinical settings.

Grimwood et al. [261] proposed the use of CycleGAN to create synthetic Endoscopic ultrasound (EUS) images from CT data, which can be used as a data augmentation strategy when EUS data is scarce.

### 6.7. Non-Contrast and Contrast-Enhanced Image

Non-contrast-enhanced medical imaging entails the acquisition of images without the administration of contrast agents. This imaging modality relies on the inherent contrast of natural tissues to visualize anatomical structures and identify potential abnormalities. Non-contrast imaging is commonly employed for routine screenings, initial assessments, and follow-up examinations. It is considered a safer option for patients with contraindications or allergies to contrast agents. Nonetheless, there are scenarios where non-contrast imaging may be limited, and the use of contrast-enhanced imaging could offer additional diagnostic insights.

Contrast-enhanced medical imaging involves the introduction of contrast agents, typically through intravenous administration, to enhance the visualization of specific anatomical structures or physiological processes [262]. These contrast agents contain substances that augment the visibility of blood vessels, organs, tumors, or regions with altered perfusion. Contrast-enhanced imaging proves particularly valuable in situations where non-contrast imaging may not provide adequate diagnostic information. For instance, in contrast-enhanced CT scans, iodine-based contrast agents are intravenously injected to accentuate blood vessels, tumors, and regions with abnormal blood flow, thereby improving the detection and characterization of lesions, vascular anomalies, and tumors. In contrast-enhanced MRI, gadolinium-based contrast agents are commonly utilized to enhance the visualization of blood vessels, brain tumors, and areas with compromised blood–brain barrier integrity, making it indispensable in neuroimaging and the diagnosis of conditions such as multiple sclerosis [263].

Contrast-enhanced imaging plays a pivotal role in diagnosing and characterizing various medical conditions, including tumors, vascular irregularities, inflammation, and ischemia. It furnishes critical insights into the dynamic behavior of tissues, enhancing the specificity and sensitivity of imaging investigations. However, certain patients may not be eligible for contrast agent injections due to various factors. To address this challenge, generative models can be employed to translate non-contrast-enhanced images into contrast-enhanced images [264].

In this section, we provide an overview of research papers focused on the translation between non-contrast-enhanced images and contrast-enhanced images. Table 11 presents essential details from these studies, offering a valuable reference for further exploration of this topic.

Zhao et al. [267] introduced a Tripartite Generative Adversarial Network (Tripartite-GAN) for synthesizing contrast-enhanced MRI (CEMRI) to detect tumors without the need for contrast agent injection. The Tripartite-GAN comprises three interconnected networks—an attention-aware generator, a convolutional neural network-based discriminator, and a region-based convolutional neural network-based detector. This integrated framework facilitates the synthesis of CEMRI and tumor detection, with the generator aiding accurate tumor detection by synthesizing tumor-specific CEMRI and the detector enhancing the generator for precise CEMRI synthesis through back-propagation.

Chen et al. [274] proposed a deep learning-based approach for contrast-enhanced T1 synthesis in brain tumor patients. A 3D high-resolution FCN designed to maintain high-resolution information and aggregate multi-scale information in parallel is employed to map pre-contrast MRI sequences (T1, T2, and ADC) to CEMRI sequences. To address data imbalance between normal tissues and tumor regions, a local loss is introduced to enhance the contribution of tumor regions, resulting in improved tumor enhancement.

Ristea et al. [278] presented a novel approach for translating NCCT scans to CECT scans and vice versa. The approach, named CyTran (cycle-consistent generative adversarial convolutional transformers), is trainable on unpaired images due to the integration of a multi-level cycle consistency loss. In addition to the standard cycle-consistency loss at the image level, additional cycle-consistency losses between intermediate feature representations are proposed, enforcing cycle-consistency at multiple representation levels, and leading to superior results. To handle high-resolution images, a hybrid architecture based on convolutional and multi-head attention layers is designed.

## 7. Discussion

This review provides a comprehensive summary of prior research on the utilization of generative models in the domain of medical image analysis. Through a synthesis of relevant literature, we categorize the applications of generative models in medical image analysis into two main segments: creation and translation. Building upon the diverse application scenarios of generative models in medical image analysis, this paper organizes previous studies into categories and offers practical implementation guidelines gleaned from the lessons learned in these works.

In Section 4, we conduct a thorough review of three widely employed generative models: VAEs, GANs, and diffusion models. We outline algorithms within these generative models that have found extensive applications in the domain of medical image analysis and provide analyses thereof.

In Section 5, we present an extensive review of creation methods. Depending on the downstream tasks, we classify creation’s downstream applications into three distinct categories: classification, segmentation, and others. Among these, 27 studies focus on classification tasks, 26 studies on segmentation, and 11 studies on various other tasks. Our literature review consistently indicates that across various downstream tasks, data augmentation methods grounded in generative models consistently result in enhanced model performance, particularly when dealing with limited annotation resources.

Section 6 classifies translation methods based on the target modality. For the MRI modality, we identify 61 studies, with 42 studies primarily centered on inter-modal translation within MRI, particularly concentrated on brain images, while 19 studies encompass modalities such as CT, PET to MRI translation. Additionally, there are 77 studies for CT, 5 for X-ray, 6 for PET, and 1 for US, respectively. Furthermore, we conduct a separate analysis of 19 studies involving non-contrast-enhanced and contrast-enhanced image translations.

Our comprehensive literature review underscores the notable advancements in GANs over recent years, with the majority of translation tasks predominantly relying on GAN-based methodologies. Furthermore, the introduction of DDPM has witnessed an increasing number of diffusion models being employed for translating medical images across different modalities. The remarkable image generation capabilities of diffusion models have significantly elevated the quality of synthesized images, albeit the inherent challenge of slow inference speed remains a critical concern [34].

Given the promising and rapidly evolving nature of medical image generation research, alongside the ongoing exploration of optimal image generation algorithms, researchers are encouraged to not only fine-tune strategies and pre-trained weights but also systematically investigate self-supervised learning techniques across various categories within their medical image datasets [39]. Additionally, testing newly developed strategies on multiple datasets, ideally encompassing diverse modalities and medical imaging domains, is recommended to foster a more comprehensive understanding of their potential and limitations.

### 7.1. Implementation Suggestion

Given the rapidly evolving nature and significant practical implications of medical image generation and translation, along with the increasing prominence of diffusion models, the pursuit of an optimal medical image generative model remains an ongoing challenge. In response, we have conducted a thorough survey and detailed comparative analysis of prior research. Our goal is to offer researchers a comprehensive set of implementation guidelines to support their exploration of methodologies in medical image generation and translation.

#### 7.1.1. Unified Model or Task-Specific Model?

Unified models refer to generative architectures designed to perform multiple medical image generation tasks using a shared network structure [38,139,147]. In contrast, task-specific models are optimized for a single generation task or imaging scenario and are typically tailored to particular modalities, anatomical regions, or downstream objectives [160,161,162]. Task-specific designs can achieve strong performance within their targeted scope but may exhibit limited generalizability when applied to substantially different tasks. Despite increasing interest in unified generative frameworks, drawing definitive conclusions regarding their superiority over task-specific models remains challenging. The selection of an appropriate generative modeling strategy is influenced by multiple factors, including dataset size, imaging modality, anatomical complexity, and the requirements of downstream clinical tasks [17,34].

Within the scope of this review, only a limited number of studies have rigorously evaluated generative models across multiple imaging modalities and anatomical regions. The majority of existing work focuses on single-modality or single-organ settings, which limits the ability to assess model generalization in diverse clinical scenarios. In practice, substantial variations across imaging modalities, organs, tissues, and pathological conditions pose significant challenges for unified models, as a single architecture may struggle to capture highly heterogeneous data distributions with consistent performance. Current empirical evidence does not support a definitive preference for either unified or task-specific generative models. Instead, available studies suggest that task-specific tailoring may offer practical advantages in scenarios where imaging characteristics or anatomical structures differ substantially. However, this observation should be interpreted as a practical consideration rather than a prescriptive recommendation. Systematic benchmarking across multiple modalities and anatomical regions is required to establish clear guidelines on when unified models can effectively generalize and when task-specific designs are more appropriate.

#### 7.1.2. GAN or Diffusion Model?

Since their introduction in 2014, GANs have been widely adopted in medical image generation. Their popularity can largely be attributed to the adversarial learning paradigm, which enables GANs to generate high-fidelity images and has consistently demonstrated superior perceptual quality compared to earlier generative approaches such as VAEs and flow-based models [282]. Nevertheless, GANs are known to suffer from training instability, with performance being sensitive to hyperparameter selection, network architecture, and regularization strategies. Despite these challenges, GAN-based methods remain the most extensively used generative framework in medical image generation to date [21].

The generative modeling landscape shifted notably with the introduction of DDPMs in 2020 [52]. Diffusion models have demonstrated strong theoretical properties, including stable training dynamics and improved mode coverage, and have achieved state-of-the-art performance in several natural image generation benchmarks. Empirical studies suggest that diffusion models are capable of capturing a broader range of sample diversity compared to GANs, while maintaining high structural fidelity [283]. However, this advantage is accompanied by increased computational cost, as diffusion models typically require multiple iterative denoising steps during sampling, resulting in slower inference compared to GAN-based approaches.

This trade-off reflects a broader generative modeling dilemma, as discussed by Kazerouni et al. [34]. GANs excel at fast generation and high visual fidelity but often struggle with limited mode coverage, whereas VAEs and normalizing flows favor diversity at the expense of perceptual quality. Diffusion models aim to reconcile these competing objectives by achieving both broad mode coverage and high-quality generation. Nonetheless, their iterative sampling process remains a practical limitation, motivating ongoing research into accelerated sampling strategies and efficiency-oriented model variants [283,284].

In the context of image creation tasks, both GANs and diffusion models have been successfully applied to unconditional and conditional image generation. Existing evidence indicates that diffusion models often produce samples with improved diversity and structural consistency, particularly in complex anatomical settings [285]. In contrast, for image translation tasks, GAN-based methods continue to dominate the field. Well-established frameworks such as Pix2Pix and CycleGAN provide strong baselines for paired and unpaired translation, respectively, and have been extensively validated across a wide range of medical imaging modalities.

At present, there is no widely accepted diffusion-based baseline model that offers a comparable level of maturity and empirical validation for medical image translation tasks. Consequently, while diffusion models exhibit substantial theoretical potential and promising preliminary results, their practical superiority over established GAN-based approaches in translation scenarios has not yet been conclusively demonstrated. Comprehensive benchmarking across diverse modalities, anatomical regions, and clinical settings is required before definitive conclusions can be drawn regarding the relative merits of GANs and diffusion models in medical image generation.

#### 7.1.3. Translation with Prior Knowledge

Due to the significant differences in information content among medical images from various modalities—often being entirely distinct—the importance of incorporating prior knowledge into medical image generation tasks becomes clear. Integrating prior knowledge is a crucial step toward improving the quality, authenticity, and clinical relevance of the generated images. This incorporation serves multiple purposes, guiding the generative process and ensuring that the resulting images maintain anatomical accuracy and clinical utility [128,286].

One strategy in the integration of prior knowledge involves the design of custom loss functions, engineered specifically to impose constraints rooted in prior knowledge [162,267]. A tangible illustration of this entails the incorporation of penalties or regularization terms into the loss function. These augmentations serve to incentivize the generated images to closely adhere to known anatomical structures or established clinical guidelines.

Furthermore, a critical aspect of leveraging prior knowledge involves preprocessing the training data. This preprocessing aims to highlight or extract specific features or anatomical structures of interest. Techniques such as image segmentation, registration, and other image processing methods can be strategically applied to enhance the quality of the input dataset, thereby providing the model with more robust and informative data.

In the pursuit of fortifying the model’s capacity to leverage prior knowledge, recourse to pre-trained models or knowledge derived from related medical imaging tasks is a valuable strategy. Transfer learning emerges as a potent technique, allowing the model to glean insights from prior knowledge encoded within models trained on analogous tasks or datasets.

Recent work, such as GradXcepUNet [287], highlights the importance of incorporating explainability and prior knowledge into medical image analysis pipelines. Although GradXcepUNet is primarily designed for segmentation rather than image generation, its use of Grad-CAM to identify diagnostically salient regions provides valuable insights for generative modeling. In particular, attention maps derived from downstream tasks could be leveraged to guide generative data augmentation, ensuring that synthetic images preserve clinically critical structures.

#### 7.1.4. Paired Versus Unpaired Image Translation

Across the reviewed literature, image translation methods that rely on paired training data remain dominant. Based on our analysis of Table 6, Table 7, Table 8, Table 9, Table 10 and Table 11, the majority of studies employ paired datasets, reflecting the strong supervision signal provided by spatially aligned image pairs. Paired approaches generally demonstrate superior quantitative performance, particularly in tasks requiring precise voxel-wise correspondence, such as MRI-to-CT or multi-contrast MRI translation [17,38,115,121]. In contrast, unpaired methods are primarily adopted in scenarios where paired data are impractical or unavailable, such as cross-institutional studies or retrospective data collection. While unpaired approaches offer greater flexibility and broader applicability, they often suffer from weaker supervision and increased ambiguity in structure preservation, which may lead to inconsistent anatomical mappings [36,77]. Performance comparisons reported in the literature suggest that paired methods consistently outperform unpaired ones in terms of aware metrics, whereas unpaired methods are more sensitive to training instability and mode collapse. Nevertheless, unpaired translation remains indispensable in real-world clinical settings where strict data pairing cannot be guaranteed. Future research should explore hybrid or semi-supervised strategies that can leverage limited paired data while maintaining the scalability of unpaired approaches.

#### 7.1.5. Other Possible Optimization Strategies for Training

In addition to adversarial training, alternative optimization strategies such as gradient perturbation and knowledge distillation have been proposed to enhance model robustness [288,289]. Gradient perturbation techniques improve the robustness and generalization of generative models by introducing controlled noise during training [290]. This strategy strengthens the model’s ability to adapt to unseen data and reduces overfitting to specific training distributions [291]. Knowledge distillation optimizes medical image generation by transferring knowledge from complex teacher models to lightweight student models [292]. It enhances the model’s generalization across multiple tasks, enabling it to perform diverse medical image generation tasks such as denoising, super-resolution, and modality translation [293]. By leveraging the stable generative capabilities of the teacher model, knowledge distillation mitigates the instability issues commonly encountered during generative model training [294,295].

### 7.2. Challenges in Medical Image Creation and Translation

#### 7.2.1. Privacy Preservation and Data Protection

Given the sensitive nature of medical imaging data, privacy preservation is a critical consideration in the development and deployment of generative models. Although generative approaches are often promoted as an effective means of alleviating data scarcity through synthetic sample generation, recent studies have demonstrated that inadequately trained models may inadvertently memorize and reproduce identifiable patient information [296,297].

To address these concerns, several privacy-preserving strategies have been proposed. Differential privacy mechanisms introduce controlled noise during the training process to reduce the risk of data leakage [296,298], while federated learning enables decentralized model training across multiple institutions without direct data sharing [84]. In addition, techniques such as secure multi-party computation and encrypted model inference have been explored to further enhance data protection in collaborative medical environments [299].

However, incorporating privacy constraints typically introduces a trade-off between data utility and image fidelity. In the medical domain, where subtle anatomical structures may carry critical diagnostic significance, excessive privacy enforcement can degrade clinically relevant features [296,298]. Therefore, future research should focus on balancing privacy preservation with diagnostic integrity, particularly in high-stakes clinical applications where both data security and image quality are essential.

#### 7.2.2. Safe Deployment and Clinical Reliability

Beyond algorithmic performance, the safe deployment of generative models in clinical practice presents substantial challenges. Synthetic medical images may contain subtle artifacts or statistically plausible yet clinically implausible structures, which can potentially mislead downstream diagnostic models or clinicians if not rigorously validated [300,301].

Another critical concern is distribution shift. Generative models trained on historical datasets may fail to generalize to evolving imaging protocols, scanner upgrades, or demographic changes. Without continuous monitoring and periodic revalidation, such models risk producing outputs that are inconsistent with current clinical standards.

Furthermore, the long-term diagnostic implications of incorporating synthetic images into clinical decision-making pipelines remain largely unexplored. Prolonged reliance on generated data may introduce systematic biases or reinforce model-specific artifacts over time [301]. Consequently, rigorous longitudinal studies, post-deployment auditing mechanisms, and continuous performance assessment are essential to ensure sustained clinical safety and reliability.

#### 7.2.3. Open Problems and Research Gaps

Despite significant progress, several open challenges remain unresolved in the application of generative models to medical imaging. First, there is a lack of standardized evaluation metrics. Many existing studies rely primarily on image similarity measures that may not adequately reflect clinical utility or diagnostic relevance [301].

Second, generalization across institutions and modalities remains limited, as most models are evaluated on single-center datasets, restricting conclusions about real-world robustness and transferability [84].

Third, training stability and reproducibility continue to pose major obstacles, particularly for GAN-based and hybrid architectures that are sensitive to hyperparameter selection and training dynamics.

Fourth, integration with clinical workflows is insufficiently explored, with relatively few studies addressing how generative models can be deployed in a transparent, interpretable, and clinically acceptable manner. Finally, the long-term clinical impact of synthetic data usage, including its effects on diagnostic accuracy and clinician trust, remains poorly understood [301].

Addressing these challenges will require close interdisciplinary collaboration among machine learning researchers, clinicians, and regulatory bodies to ensure the responsible and effective adoption of generative models in medical imaging.

### 7.3. Limitations and Future Research

This review paper summarizes recent advancements in deep learning-based medical image generation methods. However, several limitations should be acknowledged. First, it was not feasible to aggregate or statistically compare the performance of different generative models due to the heterogeneity of the included studies. These studies employed diverse imaging modalities, reported varied evaluation metrics, and applied models to different downstream tasks, making direct comparisons challenging.

Second, our classification approach was unidimensional. In Section 5, we categorized studies based on downstream tasks, while in Section 6, we grouped them by target modality for image generation. This approach may not facilitate a comprehensive comparative analysis of generative model methods. Future reviews could adopt a more nuanced classification framework, such as categorizing studies by image generation techniques, which might provide deeper insights into the strengths and weaknesses of different generation methods.

Third, while diffusion models have rapidly advanced in natural image generation since 2022 [285] and are increasingly being applied to medical image generation [34], most relevant papers are currently in preprint form. Due to the absence of peer review, these preprints were excluded from this review, resulting in limited coverage of diffusion models. As these models gain traction in medical image generation, future reviews should incorporate peer-reviewed studies to provide a more comprehensive assessment.

The study selection process may also have introduced biases. Reliance on specific databases and keywords might have excluded relevant studies from other sources, such as conference proceedings and technical reports. Additionally, the focus on English-language literature and timeframe restrictions may have led to the omission of significant non-English studies or earlier research. These limitations highlight the need for broader search strategies in future reviews.

Regarding gaps in the literature, several underexplored areas were identified. For instance, the application of physics-based generative models in medical image generation has received limited attention. Similarly, research on the generalizability of generative models across different disease stages or imaging devices remains scarce. Addressing these gaps could enhance the comprehensiveness of future assessments.

Finally, several challenges were encountered during the review process. Standardizing data extraction was difficult due to variations in metrics and reporting methods across studies, which hindered direct comparisons. The heterogeneity of studies, such as differences in dataset size, quality, and evaluation metrics, further complicated the synthesis of findings. Additionally, insufficient methodological detail in some studies made it challenging to fully interpret their results and methodologies. These challenges underscore the need for more standardized reporting practices in future research.

## 8. Conclusions

Medical image generation based on deep learning is an emerging and rapidly growing field. In this review, we categorize medical image generation into two main types: creation and translation. Creation focuses on generating new images from potential conditional variables, while translation involves mapping images from one or more modalities to another, preserving semantic and informational content.

Currently, medical image generation based on deep learning primarily utilizes three models: VAE, GANs, and Diffusion models. Each of these models has distinct characteristics, and the choice among them depends on the specific requirements of the task. The diffusion model, in particular, has demonstrated outstanding performance in natural image generation, earning widespread recognition. It is reasonable to anticipate that future research will increasingly focus on medical image creation and translation using diffusion models. Exploring methods to reduce the training and inference time of diffusion models while maintaining high generation quality may represent a promising research direction in the years to come.

Through an analysis of the literature included in this review, it is evident that deep learning-based medical image creation is a powerful technique for enhancing the performance of downstream tasks. It addresses data limitations, improves generalization, reduces overfitting, and increases model robustness to diverse imaging conditions and variations. Modality translation, on the other hand, can supplement missing modalities, aiding in diagnosis and enhancing downstream tasks such as attenuation correction for PET/MRI, radiotherapy planning without CT images, and CBCT denoising. Additionally, translation between contrast-enhanced and non-contrast images primarily aims to reduce costs, both in terms of time and financial resources, while also offering the potential for more accurate diagnoses, particularly for patients with kidney diseases. Overall, medical image translation based on generative models is multifaceted, highly effective, and holds significant promise for the future.

## Figures and Tables

**Table 1:** Quantitative evaluation metrics of medical image creation.

Symbol	Name	Formula
IS	Inception Score	$IS\left(P_{g}\right)=e^{E_{x\sim P_{g}}\left[KL\left(p_{M}\left(y|x\right)||p_{M}\left(y\right)\right)\right]}$
MS	Mode Score	$MS\left(P_{g}\right)=e^{E_{x\sim P_{g}}\left[KL\left(p_{M}\left(y|x\right)||p_{M}\left(y\right)\right)\right]-KL\left(p_{M}\left(y\right)||p_{M}\left(y^{*}\right)\right)}$
MMD	Kernel Maximum Mean Discrepancy	$MMD\left(P_{r},P_{g}\right)=E_{\begin{array}{c} x_{r},x_{r}^{\prime }∼P_{r},\\ x_{g},x_{g}^{\prime }∼P_{g} \end{array}}\left[k\left(x_{r},x_{r}^{\prime }\right)-2k\left(x_{r},x_{g}\right)+k\left(x_{g},x_{g}^{\prime }\right)\right]$
WD	Wasserstein distance	$WD\left(P_{r},P_{g}\right)={inf}_{\gamma \in \Gamma (P_{r},P_{g})}E_{(x_{r},x_{g})\sim \gamma }\left[d(x_{r},x_{g})\right]$
FID	Fréchet Inception Distance	$FID\left(P_{r},P_{g}\right)=\left\|\mu_{r}-\mu_{g}\right\|+Tr(C_{r}+C_{g}-2{\left(C_{r}C_{g}\right)}^{1/2})$

**Table 2:** The studies on medical image creation for the classification task.

Paper	Model	Anatomy	Modality	Dimension
[58]	DCGAN, ACGAN	Liver	CT	2D
[59]	DCGAN, WGAN, BEGAN	Thyroid	OCT	2D
[60]	ACGAN	Limb	X-ray	2D
[30]	ICVAE	Spine, brain	Ultrasound, MRI	2D
[61]	DCGAN	Chest	X-ray	2D
[62]	-	Lung	CT	3D
[63]	PGGAN	Chest	X-ray	2D
[64]	MTT-GAN	Chest	X-ray	2D
[65]	CT-SGAN	Chest	CT	3D
[66]	COViT-GAN	Chest	CT	2D
[67]	Two-stage GAN	Liver	Ultrasound	2D
[68]	TripleGAN	Breast	Ultrasound	2D
[69]	InfoGAN	Lung	CT	2D
[70]	GAN	Chest	X-ray	2D
[71]	LSN	Brain	CT	2D
[72]	StyleGAN2	Chest	X-ray	2D
[73]	DCGAN, cGAN	Prostate	MRI	2D
[74]	TMP-GAN	Breast, pancreatic	X-ray, CT	2D
[75]	CycleGAN	Chest	X-ray	2D
[76]	PLGAN	Ophthalmology, brain, lung	OCT, MRI, CT, X-ray	2D
[77]	CUT	Chest	X-ray	2D
[78]	HBGM	Coronary	X-ray	2D
[79]	DC-GAN	Chest	X-ray	2D
[80]	MI-GAN	Chest	CT	2D
[81]	StyleGAN2	Chest	X-ray	2D
[40]	DDPM	Chest, heart, pelvis, abdomen	MRI, CT, X-ray	2D
[82]	StynMedGAN	Chest, brain	MRI, CT, X-ray	2D

**Table 3:** The studies on medical image creation for the segmentation task.

Paper	Model	Anatomy	Modality	Dimension
[86]	Two-stage GAN	Intravascular	Ultrasound	2D
[87]	SpeckleGAN	Intravascular	Ultrasound	2D
[88]	CycleGAN	Gastrocnemius medialis muscle	Ultrasound	2D
[89]	Private	-	-	2D
[90]	Pix2Pix	Bone surface	Ultrasound	2D
[31]	VAE	-	Ultrasound	2D
[91]	Pix2Pix	Prostate	MRI	2D
[86]	CG-SAMR	Brain	MRI	3D
[92]	GAN, VAE	Thyroid	Ultrasound	2D
[93]	WFT-GAN	-	-	2D
[94]	Dense GAN	Lung	CT	2D
[95]	VAE, GAN	Cardiac	MRI	3D
[96]	LEGAN	Retinal	Digital retinal images	2D
[97]	spGAN	Lung, hip joint, ovary	Ultrasound	2D
[98]	cGAN	Cardiac	MRI	2D
[99]	SR-TTT	Liver	CT	2D
[100]	Pix2Pix, CycleGAN, SPADE	Brain	MRI	2D
[101]	SPADE	Rectal	MRI	3D
[102]	Three-dimensional GAN	Lung	CT	3D
[103]	-	Lung	X-ray	2D
[104]	-	Brain	MRI	3D
[105]	DCGAN	Retinal, coronary, knee	X-ray, MRI	2D
[106]	Pix2Pix	Lung	CT	2D
[107]	-	Cheat	X-ray	2D
[108]	Pix2Pix	Lung	CT	2D
[109]	MinimalGAN	Retinal fundus	Nature	2D

**Table 4:** The studies on medical image creation for other tasks.

Paper	Model	Anatomy	Modality	Dimension	Task
[111]	DCGAN, WGAN	Brain	MRI	2D	None
[112]	MCGAN	Lung nodules	CT	3D	Object detection
[113]	SMIG	Brain glioblastoma	MRI	3D	Tumors growth prediction
[114]	InfoGAN	Fetal head	Ultrasound	2D	None
[115]	Private	Prostate	MRI	2D	Prostate Cancer Localization
[116]	DCGAN-PSO	Lung	X-ray	2D	None
[117]	U-Net	Lung nodules	X-ray	2D	Object detection
[118]	3D-StyleGAN	Brain	MRI	3D	None
[119]	CGAN, DCGAN, f-GAN, WGAN, CycleGAN	Lung	X-ray, CT	2D	None
[120]	DCGAN	Brian	MRI	2D	None
[121]	DeepAnat	Brian	MRI	3D	Neuroscientific applications

**Table 5:** Quantitative evaluation metrics of medical image translation.

Symbol	Name	Formula
MAE	Mean Absolute Error	$\frac{1}{m}\sum_{i=1}^{m}\left|y_{i}-x_{i}\right|$
MSE	Mean Squared Error	$\frac{1}{m}\sum_{i=1}^{m}{\left(y_{i}-x_{i}\right)}^{2}$
RMSE	Root Mean Squared Error	$\sqrt{MSE}$
PSNR	Peak Signal-to-Noise Ratio	$20\cdot \log_{10}\left(\frac{MAX}{RMSE}\right)$
SSIM	Structural Similarity Index	$\frac{\left(2\mu_{x}\mu_{y}+C_{1})(2\sigma_{xy}+C_{2}\right)}{\left(\mu_{x}^{2}+\mu_{y}^{2}+C_{1})(\sigma_{x}^{2}+\sigma_{y}^{2}+C_{2}\right)}$

**Table 6:** The studies on the multi-contrast MRI translation.

Paper	Dataset	Dimension	Modality Translation	Model	
				Name	Paired Image
[126]	BraTS 2015	3D	T1→FLAIR	Three-dimensional cGAN	Yes
[38]	MIDAS, IXI, BraTS	2D	T1↔T2	pGAN, cGAN	Yes, No
[127]	BraTS 2015, IXI	3D	T1→FLAIR; T1→T2	Ea-GANs	Yes
[128]	BraTS 2018	2D	T1, T2, T1ce, FLAIR (three-to-one)	Auto-GAN	Yes
[125]	ISLES 2015, BraTS 2018	2D	T1, T2, DWI; T1, T1ce, T2, FLAIR (generating the missing contrast(s))	MM-GAN	Yes
[129]	BraTS 2018	2D	T1↔T2	-	Yes
[130]	Private	2D	T1↔T2	CACGAN	No
[131]	BraTS 2018	2D	T2→(FLAIR, T1, T1ce)	TC-MGAN	Yes
[132]	BraTS 2015, SISS 2015	3D	T1→FLAIR; T1→T2	SA-GAN	Yes
[133]	BraTS 2018	2D	T1↔T2; T1↔FLAIR; T2↔FLAIR; T1, T2, FLAIR (Two-to-One)	Hi-Net	Yes
[134]	BraTS 2017, TCGA	2D	(T1ce, FLAIR)→T2	-	Yes
[135]	BraTS 2018	2D	T1, T2, T1ce, FLAIR (generating the missing contrast(s))	-	Yes
[136]	BraTS 2015	2D	T1→FLAIR; T1→T2	EP-IMF-GAN	Yes
[137]	HCP 500	2D	B0→DWI; B0, T2→DWI; B0, T1, T2→DWI	-	Yes
[138]	Private, IXI	2.5D	T1→T2	-	Yes
[139]	IXI	2D	T2↔PD	DiCyc	No
[140]	BraTS 2015	2D	T1↔T2	-	No
[141]	IXI, BraTS 2019	2D	Unified model	Hyper-GAN	Yes
[142]	IXI, ISLES	2D	T1↔T2; T1↔PD; T2↔PD; T1↔FLAIR; T2↔FLAIR; T1, T2, PD (two-to-one); T1, T2, FLAIR (two-to-one)	mustGAN	Yes
[143]	BraTS 2015	2D	T1, T1ce→FLAIR; T1, T2→FLAIR; T1, T1ce→T2	LR-cGAN	Yes
[144]	BraTS 2018	3D	T1, T2, T1ce, FLAIR (generating the missing contrast(s))	-	Yes
[145]	ADNI	2D	T1→CBV	DeepContrast	Yes
[146]	Private	2D	PD↔T2	-	No
[147]	IXI, BraTS	2D	T1, T2, PD (two-to-one); T1, T2, FLAIR (two-to-one) PD↔T2; FLAIR↔T2	ResViT	Yes
[94]	IXI	2D	T2→PD	TR-GAN	Yes
[148]	BraTS2019	3D	T1, T2, T1ce, FLAIR (generating the missing contrast(s))	CoCa-GAN	Yes
[149]	-	2D	T2↔DWI	CICVAE	No
[150]	BraTS2019	2D	T1→T2	NEDNet	Yes
[151]	BraTS, Brain, SPLP	2D	T1↔T2	Bi-MGAN	No
[152]	IXI, vivo brain dataset	2D	T1, T2, PD (two-to-one); T1, T2, T1ce, FLAIR (three-to-one)	ProvoGAN	Yes
[153]	BraTS 2015, IXI	2D	T1↔T2; T1→FLAIR; T2→FLAIR; T2↔PD	D2FE-GAN	Yes
[154]	dHCP, BCP	3D	T1↔T2	PTNet3D	Yes
[155]	BraTS 2018	2D	T1↔FLAIR; T1↔T2	DualMMP-GAN	No
[156]	BraTS 2020, ISLES 2015, CBMFM	2D	T1, T2, FLAIR, T1ce (three-to-one); T1, T2, FLAIR, DWI (three-to-one)	AE-GAN	Yes
[157]	Private	2D	T1→DWI; T2→DWI; T1, T2→DWI; T1→FLAIR; T2→FLAIR; T1, T2→FLAIR	GAN	Yes
[158]	IXI, BraTS 2021	2D	T1, T2, PD; T1, T1ce, T2, PD (generating the missing contrast(s))	MMT	Yes
[42]	BraTS, IXI	2D	T1↔T2; T1↔PD; T2↔PD; T1↔FLAIR; T2↔FLAIR	SynDiff	No
[159]	BraTS 2018, IXI	2D	PD, MRA, T2 (two-to-one)	LSGAN	No
[160]	BraTS 2018, IXI	2D	PD, MRA, T2 (two-to-one)	-	Yes
[161]	Private	2D	T1, T2, ADC, T1ce, FLAIR→CBV	-	Yes
[162]	MRM NeAt Dataset; Private	2D	T1↔T2	MouseGAN	No

**Table 7:** The studies on image translation to MRI from other modalities.

Paper	Origin Modality	Anatomy	Dataset	Dimension	Model	
					Name	Paired Image
[163]	CT	Lung	NSCLC	2D	CycleGAN	No
[164]	CT	Brain	Private	2D	-	Yes
[165]	CT	Pelvis	Private	3D	CycleGAN	No
[166]	CT	Abdomen	Private	2D	Pix2Pix	Yes
[167]	CT	Brain	ADNI	3D	-	Yes
[168]	CT	Brain, abdomen	Private	2D	BPGAN	Yes
[169]	CT	Liver	CHAOS	2D	TarGAN	Yes
[170]	CT	Pelvis	Private	3D	CycleGAN	No
[171]	CT	Head and neck	Private	2D	-	Yes
[93]	CT	Abdomen	CHAOS	2D	WFT-GAN	No
[146]	CT	Brain	Private	2D	-	No
[172]	CT	Prostate	Private	2D	PxCGAN	Yes
[173]	CT	Brain	From [174]	2D	DC-CycleGAN	No
[175]	CBCT	Prostate	Private	3D	CycleGAN	Yes
[176]	CBCT	Brain	Private	3D	TGAN	Yes
[177]	PET	Brain	Private	2D	-	Yes
[178]	PET	Brain	ADNI	3D	E-GAN	Yes
[179]	Ultrasound	Brain	INTERGROWTH-21st, CRL	2D	-	No

**Table 8:** The studies on image translation to CT from other modalities.

Paper	Origin Modality	Anatomy	Dataset	Dimension	Model	
					Name	Paired Image
[187]	CBCT	Nasopharyngeal carcinoma	Private	2D	U-Net	Yes
[188]	CBCT	Head and neck	Private	2D	CycleGAN	No
[189]	CBCT	masseter	Private	2D	CycleGAN-based	No
[180]	CBCT, MRI	Head and neck	Private	2D	U-Net	Yes
[190]	CBCT	Head and neck	Private	2D	U-Net	Yes
[191]	CBCT	Head and neck	Private	2D	USsCTU-net	No
[192]	CBCT	Head and neck, pelvic	Private	2D	Cycle-RCDC-GAN	Yes
[176]	CBCT, MRI	Brain	Private	3D	TGAN	Yes
[193]	CBCT	Head and neck, pelvic	Private	2D	DCC-GAN	No
[194]	CBCT	Brain	Private	2D	CGAN	Yes
[195]	CBCT	Abdomen	Private	2D	CycleGAN	No
[186]	CBCT	Lung	Private	2D	MURD	No
[196]	NAC-PET	Whole body	Private	3D	CycleGAN	No
[197]	NAC-PET	Whole body	Private	2D	Wasserstein GAN	Yes
[198]	PET	Whole body	Private	2D	U-Net	Yes
[199]	PET	Animal	Private	2D	-	Yes
[146]	PET, MRI	Brain, whole body	Private	2D	-	No
[200]	X-Ray	Lung	LIDC-IDRI	2D-3D	X2CT-GAN	Yes
[201]	X-Ray	Lung	PadChest	2D-3D	X2CT-GAN	Yes
[202]	MRI	Brain	Private	2D	U-Net	Yes
[203]	MRI	Pelvis	Private	2D	Pix2Pix	Yes
[204]	MRI	Brain, pelvis	ADNI, Private	3D	-	Yes
[205]	MRI	Brain, prostate	Private	3D	DECNN	Yes
[206]	MRI	Whole body	Private	2D	CycleGAN	No
[207]	MRI	Prostate	Private	2D	U-Net, GAN	Yes
[208]	MRI	Pelvis	Private	3D	Dense-Cycle-GAN	No
[209]	MRI	Liver	Private	3D	CycleGAN	No
[210]	MRI	Brain	[211]	3D	hGAN	No
[212]	MRI	Pelvis	Private	2D	Pix2PixHD	Yes
[128]	MRI	Brain	ADNI	2D	Auto-GAN	Yes
[167]	MRI	Brain	ADNI	3D	-	Yes
[213]	MRI	Brain	Private	2D	Attention-GAN	Yes
[214]	MRI	Pelvis	Private	2D	-	Yes
[215]	MRI	Liver	Private	2D	U-Net	Yes
[216]	MRI	Brain	Private	2D	U-Net	Yes
[217]	MRI	Lumbar spine	SpineWeb	3D	CycleGAN	No
[168]	MRI	Brain, abdomen	Private	2D	BPGAN	Yes
[184]	MRI	Brain, abdomen	Private, CHAOS	2D	SC-CycleGAN	No
[218]	MRI	Brain	Han et al. [112] and the JUH dataset	2D	uagGAN	Yes
[219]	MRI	Lumbar Spine	Private	2D	CycleGAN	No
[169]	MRI	Liver	CHAOS	2D	TarGAN	Yes
[220]	MRI	Pseudo	Private	2D	U-Net, GAN	Yes
[221]	MRI	Abdomen	Private	2D	SA-GAN	Yes
[222]	MRI	Pelvis, thorax, abdomen	Private	2.5D	CycleGAN	No
[223]	MRI	Head and neck	Private	3D	Label-GAN	Yes
[224]	MRI	Head and neck	Private	2D	Multi-Cycle GAN	No
[225]	MRI	Abdomen	Private	2D	-	Yes
[171]	MRI	Head and neck	Private	2D	-	Yes
[139]	MRI	Brain	IXI, MA^3^RS	2D	DiCyc	Yes
[226]	MRI	Brain	Private	2D	-	No
[93]	MRI	Abdomen	CHAOS	2D	WFT-GAN	No
[227]	MRI	Brian	Private	3D	-	Yes
[228]	MRI	Head and neck	Private	2D	-	Yes
[147]	MRI	Pelvis	Private	2D	ResViT	Yes
[229]	MRI	Brain	RIRE	2D	GCG U-Net	Yes
[230]	MRI	Head	RIRE	2D	U-Net_E-SGA_, cWGAN_E-SGA_	Yes
[231]	MRI	Head	Private	3D	ResUNet	Yes
[232]	MRI	Abdomen	Private	2D	U-Net, cGAN	Yes
[233]	MRI	Brain	Private	2D	CycleGAN	Yes
[234]	MRI	Brain	Private	3D	cGAN	Yes
[235]	MRI	Pelvis	Gold Atlas	2D	Diffusion	Yes
[236]	MRI	Brain	GKRS	2D	Pix2Pix	Yes
[237]	MRI	Brain	Atlas project	2D	Pix2Pix	Yes
[238]	MRI	Pelvis	VMAT	3D	MD-CycleGAN	No
[156]	MRI	Brain	CBMFM	2D	AE-GAN	Yes
[239]	MRI	Brain	Private	2D	CycleGAN	No
[240]	MRI	Brain	Private	2D	AMSF-Net	Yes
[241]	MRI	Abdomen	CHAOS	2D	SSA-Net	No
[42]	MRI	Pelvis	Private	2D	SynDiff	No
[242]	MRI	Abdomen	Private	2D	Pix2Pix	Yes
[37]	MRI	Brain	ABCs	2.5D	DU-CycleGAN	No
[243]	MRI	Brain	From [173]	2D	DC-cycleGAN	No
[244]	MRI	Brain	MedPix, Private	2D	MSE-Fusion	Yes
[245]	MRI	Pelvis	From [246]	2D	RTCGAN	Yes
[247]	MRI	Abdomen	Private	3D	QACL	Yes
[248]	MRI	Head and neck	Private	2D	CMSG-Net	Yes

**Table 9:** The studies on medical image translation to X-Ray from other modalities.

Paper	Origin Modality	Anatomy	Dataset	Dimension	Model	
					Name	Paired Image
[249]	DRR	Chest	JSRT, NIH	2D	TD-GAN	No
[250]	CBCT	Head	CQ500	2D	Pix2Pix	Yes
[251]	CT	Chest	LIDC-IDRI, TBX11K	2D	XraySyn	No
[252]	CT	Chest	CheXpert	2D	CT2CXR	No
[253]	X-ray	Chest	LIDC-IDRI	2D	DL-GIPS	Yes

**Table 10:** The studies on image translation to PET from other modalities.

Paper	Origin Modality	Anatomy	Dataset	Dimension	Model	
					Name	Paired Image
[257]	MRI	Brain	ADNI	3D	-	Yes
[258]	MRI	Brain	ADNI	2D	CL-GAN	Yes
[254]	MRI	Brain	ADNI	3D	BMGAN	Yes
[259]	MRI	Brain	ADNI	3D	BPGAN	Yes
[256]	CT	Liver	Private	2D	FCN-GAN	Yes
[146]	CT	Whole body	Private	2D	-	No

**Table 11:** The studies on the translation between the NC and CE images.

Paper	Modality	Translation	Anatomy	Dataset	Dimension	Model	
						Name	Paired Image
[265]	MRI	NC to CE	Brain	IXI	2D	Steerable GAN	Yes
[266]	MRI	NC to CE	Cardiac	CycleGAN	2D	MS-CMRSeg	No
[267]	MRI	NC to CE	Liver	Private	2D	Tripartite-GAN	Yes
[268]	MRI	NC to CE	Brain	Private	3D	V-net	Yes
[269]	MRI	NC to CE	Ankylosing spondylitis	Private	2D	AMCGAN	Yes
[270]	MRI	NC to CE	Liver	Private	2D	Pix-GRL	Yes
[271]	CT	NC to CE	Aorta	Private	2D	Cascade GAN	Yes
[272]	CT	NC to CE	Aorta	Private	2.5D	aGAN	Yes
[273]	MRI	NC to CE	Brain	Private	3D	BICEPS	Yes
[274]	MRI	NC to CE	Brain	Private	3D	-	Yes
[275]	CT	NC to CE	Liver	Ircadb, Sliver07, LiTS	2D	-	Yes
[276]	CT	NC to CE	Cardiac	Private	2D	Pix2Pix	Yes
[277]	MRI	NC to CE	Breast	Private	2D	TSGAN	Yes
[39]	CT	Mutual synthesis	Lung	Private	3D	Pix2Pix	Yes
[278]	CT	Mutual synthesis	Lung	Coltea-Lung-CT-100W	2D	CyTran	No
[279]	CT	NC to CE	Kidney	Private	2D	CycleGAN	No
[280]	CT	NC to CE	Lung	LIDC-IDRI, EXACT09, CARVE14, PARSE	3D	CGAN	No
[281]	CT	NC to CE	Abdomen	CHAOS, Private	3D	UMTL	Yes

## Data Availability

Data sharing is not applicable to this article as no datasets were generated or analyzed during the current study.

## Abbreviations

CBCT	Cone Beam Computed Tomography	MRA	Magnetic Resonance Angiography
CT	Computed Tomography	MRI	Magnetic Resonance Imaging
DM	Diffusion Model	PD	Proton density image
DWI	Diffusion-Weighted Image	PET	Positron Emission Tomography
FID	Fréchet Inception Distance	PSNR	Peak Signal-to-Noise Ratio
FLAIR	Fluid-Attenuated Inversion Recovery	RMSE	Root Mean Square Error
GAN	Generative Adversarial Network	SSIM	Structural Similarity Index
IS	Inception Score	T1w	T1-weighted image
MAE	Mean Absolute Error	T2w	T2-weighted image
MMD	Maximum Mean Discrepancy	US	Ultrasound imaging
MS	Mode Score	VAE	Variational Autoencoder
MSE	Mean Squared Error	WD	Wasserstein distance
